# Silver-Loaded Chabazite in Ethanol-to-Hydrocarbon Process—*Operando* FT-IR and UV-Vis Spectroscopic Studies

**DOI:** 10.3390/molecules29061207

**Published:** 2024-03-08

**Authors:** Karolina A. Tarach, Anna K. Walczyk, Agata Kordek, Oliwia Rogala, Kinga Góra-Marek

**Affiliations:** Faculty of Chemistry, Jagiellonian University in Kraków, Gronostajowa 2, 30-387 Kraków, Polandagata.kordek@student.uj.edu.pl (A.K.); oliwia.rogala@doctoral.uj.edu.pl (O.R.); kinga.gora-marek@uj.edu.pl (K.G.-M.)

**Keywords:** chabazite, silver, ethanol, *operando*, FT-IR, UV-vis, spectroscopy, catalysis

## Abstract

The ethanol dehydration process is studied regarding protonic and Ag-loaded chabazite zeolite in advanced FT-IR and UV-vis *operando* spectroscopic studies with simultaneous mass spectroscopy and gas chromatography analyses of products. The spectroscopic investigation provides information on the species formed on the surface of catalysts, while mass spectrometry and gas chromatography methods identify the desorbed products. These studies are also supported by spectroscopic, chromatographic, and thermogravimetric analyses of coke species formed over the catalyst’s surface during ethanol conversion. The Ag-chabazite catalyst shows higher selectivity for ethylene and propylene; the slower formation of coke species; and, thus, a longer lifetime.

## 1. Introduction

Hydrocarbons such as ethylene and propylene are commonly used in the petrochemical industry but are predominantly derived from non-renewable sources. Given their limited supply, there is a growing need to develop sustainable processes that use renewable sources for ethylene and propylene production. Chemical catalysts play a crucial role in creating sustainable processes. The conversion of ethanol into hydrocarbons (ETH) is an important example [1]. Ethanol can be easily derived from bioresources such as the fermentation processes of sugars or forestry and municipal organic waste [2,3].

Zeolites have proven to be prominently useful catalysts in converting ethanol into hydrocarbons, specifically, propylene [3,4]. The catalytic properties of zeolites vary depending on the structural features, such as the size and arrangement of channels and pores and the strength of acid sites. It is, therefore, essential to cover various parameters of zeolites in studying ethanol conversion. H-ZSM-5 (Si/Al = 40) has been found to be very efficient in propylene production from ethanol; however, fast deactivation was observed due to dealumination [3]. Further, the moderately acidic sites are the most active in ethanol conversion [5]. A zirconium catalyst modified by yttrium is very effective in the highly selective conversion of ethanol into propylene. Yttrium addition to ZrO_2_ increases the specific surface area and the number of active sites. The conversion of ethanol into ethylene is expected to proceed over acidic centres, while over basic centres, ethanol indirectly converts into propylene [6]. Thus, it is crucial to evaluate the role of metal-derived sites in ethanol conversion into propylene. Metal-exchanged zeolites have both Brønsted acid sites and Lewis acid functionality, which enhance their catalytic properties. It has been shown that Ag^+^ cations can function as Lewis acid sites that are capable of hydrogen abstraction and, hence, methane C-H bond activation [7,8].

Silver-modified zeolites are particularly interesting because of the possibility of modifying metal aggregation and the oxidation state within a controlled and relatively wide range. Besides isolated cations, various forms of silver can be present, such as stabilised positively charged clusters, Ag_m_^δ+^ and/or Ag_n_^0^, as well as nanoparticles (NPs). Naturally, the catalytic activity depends on the type of silver centres zeolites possess [9]. The positively charged Ag_n_^δ+^ clusters in Ag-Y, Ag-A, and Ag-ZSM-5 zeolites can catalyse the conversion of methane into propylene in the presence of ethylene [10]. Plenty of silver catalysts, especially Ag-exchanged zeolites, can catalyse many other essential reactions because of their unique properties, caused by a combination of redox and acidic sites, for example, the selective aromatisation of alkanes, alkenes, and methanol or the reduction of unwanted nitrogen oxides [11,12].

Considering that silver active sites enable the conversion of organic compounds and are relatively simple to introduce, it is worth exploring silver-modified zeolites as catalysts in the ETH process. Ag-zeolites might provide desirable features such as high activity and adjusted selectivity. The ETH process is known to proceed via the hydrocarbon pool (HCP) mechanism. For methanol conversion, the HCP is autocatalytic; thus, the conversion rate increases when a small concentration of products is present; this is continued until the steady state is achieved. Then, olefin-based and aromatic-based cycles co-occur in the catalyst’s pores, with preferences controlled by the pore’s architecture. In the case of ethanol, ethylene or diethyl ether is formed directly from ethanol during dehydration. However, the later steps are less discussed in the literature. *Operando* FT-IR and UV-vis spectroscopic studies of ethanol conversion over protonic and silver-modified chabazite zeolite might provide valuable insights into the hydrocarbon pool mechanism.

## 2. Results and Discussion

### 2.1. Structural and Textural Characterisation of Protonic and Ag-Loaded Chabazite Zeolites

**Figure 1:**
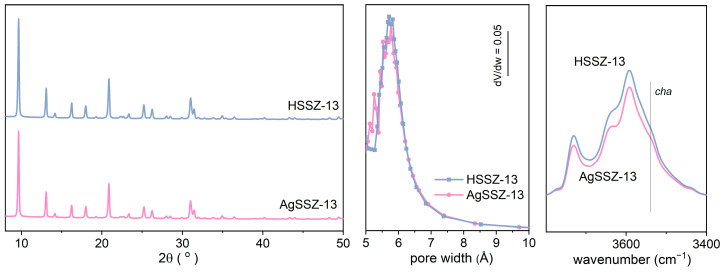
(**left**) X-ray diffraction pattern, (**middle**) differential micropore volume plot, and (**right**) IR spectra of OH group region for the studied zeolites.

The structural and textural properties of HSSZ-13 and AgSSZ-13 zeolite provide information on the studied samples’ high crystallinity and microporous characteristics (Table 1). The introduction of Ag into the SSZ-13 zeolite structure does not strongly affect its crystallinity. The presence of Ag crystalline phases is not confirmed (Figure 1); thus, the dispersion of Ag clusters is expected to be very high. Our chemical analysis shows that the Si/Al ratio does not change after the ion exchange procedure, and the Ag content is 1.6 wt%. Low-temperature nitrogen sorption shows that textural parameters are unaffected by the ion exchange procedure, and no significant drop in micropore volume is found. A differential micropore volume plot shows a slight shift in micropore width to lower values after silver introduction. The FT-IR spectra of the Si(OH)Al groups confirm that, after ion exchange, the protonic sites are substituted by Ag^+^ sites, and silver is located mainly in micropores. Thus, it might be anticipated that the low loading of silver does not significantly affect the volume of micropores even though the silver ions are expected to be located inside the micropores, most probably in spacious chabazite cages [9].

### 2.2. Operando UV-Vis and FT-IR Spectroscopic Studies Supported by Mass Spectrometry and Gas Chromatography Results—Ethanol Conversion into Ethylene and Propylene

The protonic and silver-exchanged catalysts are evaluated with *operando* UV-vis and FT-IR spectroscopic studies with a simultaneous analysis of products with gas chromatography and mass spectrometry. The applied WHSV (weight hourly space velocity) values for the *operando* UV-vis (14 h^−1^) and FT-IR (7 h^−1^) spectroscopic studies are different; as for the proper S/N ratio of the FT-IR spectra, it was necessary to reduce it.

UV-vis spectroscopy enables the differentiation of neutral and cationic species, such as alkyl-substituted benzenes, cyclopentadienes, benzenium ions, and cyclopentadienium ions. The neutral methylbenzenes are identified by bands of around 230 nm. In comparison, methylbenzenium ions are represented by a band at wavelengths longer than 320 nm. At the same time, the position of the former is independent of the number of methyl groups. For the latter, a bathochromic shift is observed with an increasing number of methyl groups [13]. Further, the bands in the 275–310 nm region are assigned to acyclic monoenyl cations and alkyl-substituted cyclopentenyl cations. The band position depends on the number and location of methyl groups on the cyclopentenyl cation [13,14]. Methanol conversion over ZSM-5 zeolite followed by UV-vis spectroscopy allows us to assign bands at 275, 315, and 375 nm to neutral aromatics, monoenylic cations, and dienylic cations, respectively [15]. Bands at 280, 320, 380, and 430 nm were found during methanol conversion over H-SAPO-34 with an assignment to neutral aromatics and monoenylic, dienylic, and trienylic cations, respectively [16]. Larger polycyclic aromatic species, such as methylnaphthalenes, were identified on H-beta zeolite; the cationic forms of 1,4-dimethylnaphthalene and 2,3-dimethylnaphthalene show bands at 381 nm. However, polycyclic species with at least two conjugated rings have also been identified by bands above 400 nm [13].

UV-vis spectra gathered during *operando* studies of ethanol conversion (WHSV = 14 h^−1^) over HSSZ-13 and AgSSZ-13 samples at 325 °C are presented in Figure 1. On the surface of protonic HSSZ-13, the zeolite conversion of ethanol in the first 25 min of reaction leads to the development of species represented by bands at 218, 254, and 300 nm. The band at 254 nm increases only at the very beginning of the reaction. The ethanol flow during the reaction course leads to the formation of a 300 nm band, which is later transformed into bands at 274, 330, 357, and 410 nm. These bands steadily increase in intensity until the reaction reaches 120 min. Mass spectrometry informs us of the population of the *m*/*z* of fragmentation ions characteristic of derived reaction products desorbed from the catalyst surface. The highest intensity is observed for *m*/*z* values at 45, 31, 28, and 18. The dominant intensity of the *m*/*z* value signals can be found for 46–39, 32–15, and 19–14. Further, trace signals of fragmentation ions with 74, 59, 56, and 55 *m*/*z* values can also be found; the latter two are only observable before 50 min of reaction has passed. The gas chromatography analysis shows that ethanol is converted into ethylene over protonic zeolite, with a conversion drop from 45% to 18% during the 120 min reaction, and the selectivity to ethylene increases from 90% to 95%. Other observed products are propane; propylene; and, initially, higher hydrocarbons (C_4+_). The Ag-exchanged chabazite zeolite provides different characteristics under reaction conditions. UV-vis bands at 202, 225, 254, and 300 nm are initially observed, and with reaction time, the species represented by the band at 300 nm are transformed into ones described by only two bands, 330 and 410 nm. The band at 254 nm grew constantly during the reaction course. No band at 274 nm can be observed for the Ag-exchanged sample. Also, the intensities of all regarded bands are distinctly lower for AgSSZ-13 than for HSSZ-13 zeolite—the introduction of silver results in a different distribution of ethanol conversion products. The mass spectroscopy analysis shows that the *m*/*z* values of the fragmentation ions at 55 and 56 can be found at later stages of the reaction for silver-exchanged sample than for the protonic sample. The gas chromatography analysis confirms the higher stability of Ag-catalysts as a conversion drop from 46% to 25% is observed, whereas selectivity to the ethylene and propylene was close to 98%.

During the first minutes of ethanol conversion over the studied zeolites, it might be anticipated that neutral olefinic and aromatic species will be formed (the bands below 220 nm and at 254 nm). Further, alkyl-substituted cyclopentenyl cations can be formed at the next step (band at 300 nm); for instance, the tetramethyl(n-propyl)cyclopentenyl cation can be identified on H-MOR at 303 nm [13]. The alkyl-substituted cyclopentenyl cations are then transformed into neutral aromatics (a band at 274 nm) and alkyl-substituted benzenium ions (bands at 330 and 357 nm). Simultaneously, naphthalene is formed and represented by a 410 nm band. The transformation of alkyl-substituted cyclopentenyl cations into other species over AgSSZ-13 zeolite is less pronounced. Similarly, the formation of naphthalene represented by the 410 nm band is also limited. This effect is accompanied by the longer stability of the AgSSZ-13 catalyst during ethanol conversion. Thus, it can be concluded that the alkyl-substituted cyclopentenyl cation species represented by the 300 nm band are crucial for the hydrocarbon pool (HCP) mechanism, driving the formation of propylene and higher hydrocarbons. The HCP primarily forms propylene and C_4+_ products, while ethylene is expected to be created from ethanol dehydration [17]. Thus, changes were observed in selectivity during the reaction course; selectivity to ethylene increases for protonic zeolite and decreases for silver-loaded zeolite in form with respect to different HCP stability over the studied catalysts. For an autocatalytic course in the reaction of the HCP process, the alkyl-substituted cyclopentenyl cation species represented by the band at 300 nm seem crucial. The changes in the selectivity of the catalysts can also be observed as trace signals on the mass spectrum, where C_4=_ (*m*/*z* = 55 and 56). The protonic zeolite forms C_4+_ hydrocarbons at the beginning of the reaction course, while silver-loaded zeolite forms them in later stages. The trace formation of diethyl ether can also be observed on the mass spectrum (*m*/*z* = 74) and seems to be constant over the reaction course; however, it is extremely low (<0.5%).

**Figure 2:**
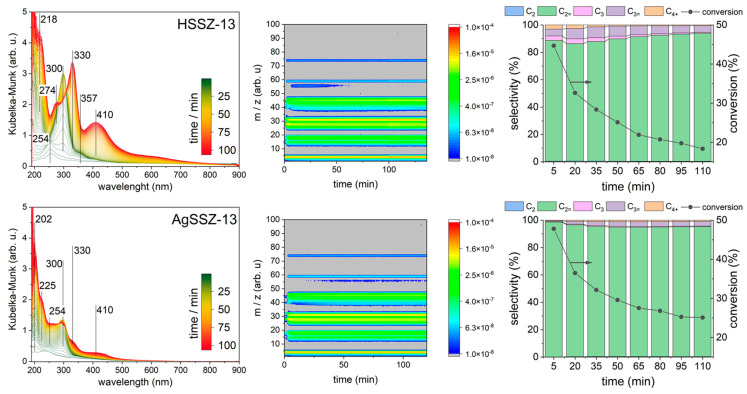
(**left**) UV-vis spectra, (**middle**) mass spectrometry results, and (**right**) gas chromatography selectivity (**left axis**) and conversions (**right axis**) collected during ethanol conversion over studied samples. The legend for the reaction products is presented in the heading.

Operando FT-IR&GC&MS studies of ethanol conversion (WHSV = 7 h^−1^) over protonic HSSZ-13 and AgSSZ-13 zeolite were performed at 325 °C. The FT-IR spectra registered for HSSZ-13 zeolite show the extensive development of bands in the 1700–1300 cm^−1^ region (Figure 2). The highest contribution can be found for the band at 1602 cm^−1^ with accompanying bands at 1505, 1470, and 1374 cm^−1^. The FT-IR spectra registered for the AgSSZ-13 catalyst were far less intensive with comparable contributions from bands located at 1648, 1617, and 1470 cm^−1^ and accompanying bands at 1675, 1566, 1505, 1448, 1418, and 1372 cm^−1^. Notably, the bands 1470 and 1448 cm^−1^ develop at a very early reaction stage, and the band located at higher wavenumbers develop later. The mass spectrometry results provide information showing that, for the studied zeolites, the signal from the fragmentation ions of the *m*/*z* values at 31, 28, and 18 are of the highest intensity, and the signals at the 46–39, 32–25, and 19–14 *m*/*z* value ranges are significantly higher than the others. The trace signals of the *m*/*z* fragmentation ions differ between the protonic and silver-loaded zeolite; in the former catalyst, 74, 70, 59, 56, 55, 53, and 51 are found, while in the latter, only 74, 59, 56, and 55 are present. The gas chromatography results show that the silver-loaded zeolite has high selectivity to ethylene and propylene; however, when WHSV = 7 h^−1^ is applied, the protonic zeolite shows higher conversion than silver-loaded chabazite.

The FT-IR spectra registered during ethanol conversion over HSSZ-13 and AgSSZ-13 zeolite differed strongly. A greater accumulation of species during the reaction can be seen in HSSZ-13 zeolite, which is related to the extensive formation of species involved in the HCP in chabazite cages. This is confirmed by the instant evolution of propylene and C_4+_ hydrocarbons after contacting the HSSZ-13 catalyst with ethanol. For AgSSZ-13, the observed IR bands are less intensive, and initially, only ethylene is formed. The intensive band located at 1602 cm^−1^ for protonic zeolite overwhelms the spectrum. While other bands lose impact after 30 min of reaction, the band at 1602 cm^−1^ constantly increases its intensity. The conversion stabilises after 30–40 min of reaction over HSSZ-13, as seen in the gas chromatography results. Thus, it might be anticipated that the 1602 cm^−1^ band species are not essential for the ongoing HCP process, at least not for propylene production. The bands at 1602 and 1617 cm^−1^ might be tentatively assigned to aromatic species as highly methylated (ethylated) benzenium cations. The higher intensity of the 1470 cm^−1^ band in AgSSZ-13 zeolite implies that, besides aromatic species (1617 cm^−1^), higher olefins are formed inside zeolite micropores when Ag is present. This is also confirmed by bands at 1675 and 1648 cm^−1^, which should be assigned to long-chain α-olefins formed inside micropores of AgSSZ-13 zeolite. Mass spectroscopy and gas chromatography showed that HSSZ-13 zeolite’s selectivity to ethylene increases with reaction time, while for AgSSZ-13 zeolite, the trend is the opposite. Ethylene is formed during dehydration, even over the relatively weak acidic centres. Thus, the acid sites in the HSSZ-13 zeolite undergo partial deactivation. The AgSSZ-13 showing increased selectivity to propylene is expected to persist in the autocatalytic mode of the HCP mechanism with only minor deactivation. Mass spectrometry provides additional insights into the mechanism of ethanol conversion. The observed fragmentation ion of *m*/*z* = 70 might originate from divinyl ether formed over the acid sites of HSSZ-13 during the first 25 min of reaction in trace amounts (not detectable with gas chromatography). After 25 min of reaction, fragmentation ions from diethyl ether (*m*/*z* = 74, 59) can be seen. Trace amounts of diethyl ether are also formed over AgSSZ-13 zeolite; however, divinyl ether cannot be found.

The *operando* FT-IR spectroscopic results provide additional insights into the products formed and accumulated inside the chabazite cages and on the surface of zeolite grains. The band at 1505 cm^−1^ in the methanol-to-hydrocarbon process is assigned to alkyl-substituted cyclopentenyl cations [13,14] that can also be identified on UV-vis spectra. However, other protonated five-membered ring cations stemming from xylenes described by the band at ca. 1485 cm^−1^ have also been proposed as intermediates in the ethanol-to-hydrocarbon process [18,19]. The latter’s presence cannot be excluded as the complex bands in regions 1510–1460 cm^−1^ are formed during ethanol conversion. The higher intensity of these bands for AgSSZ-13 also signifies the accumulation of hydrocarbons with an increased number of -CH_2_- units.

**Figure 3:**
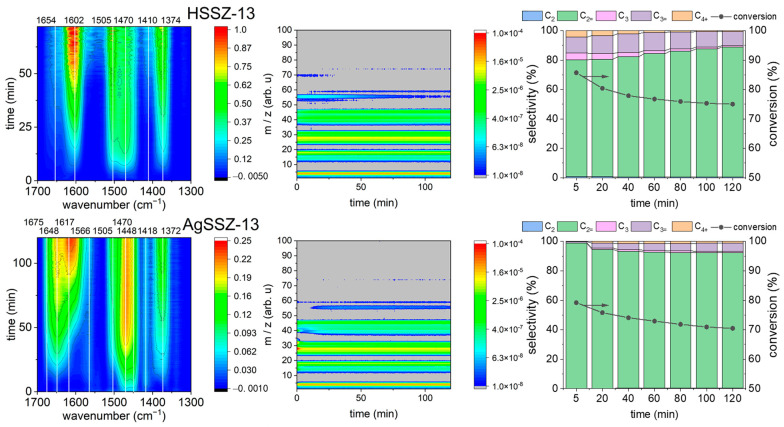
(**left**) Top-down projection of FT-IR spectra, (**middle**) mass spectrometry results, and (**right**) gas chromatography selectivity and conversions collected during ethanol conversion over studied samples.

It is worth noting that even a small amount of silver introduced into the zeolite structure has a significant impact on the catalyst’s conversion and selectivity, as well as its lifetime. An analysis of the intensity of the Si(OH)Al group bands indicates that protons are replaced by Ag^+^ ions in a number corresponding to the degree of ion exchange and that Ag^+^ ions are located in chabazite cages (Figure 1). This slight local decrease in the number of proton centres may limit the extent of subsequent bimolecular reactions, preventing deactivation and, thus, promoting the ethanol dehydration process for the AgSSZ-13 sample (as shown in Figure 2 and Figure 3).

### 2.3. Coke Analysis via GC-MS and TG-IR Methods

**Figure 4:**
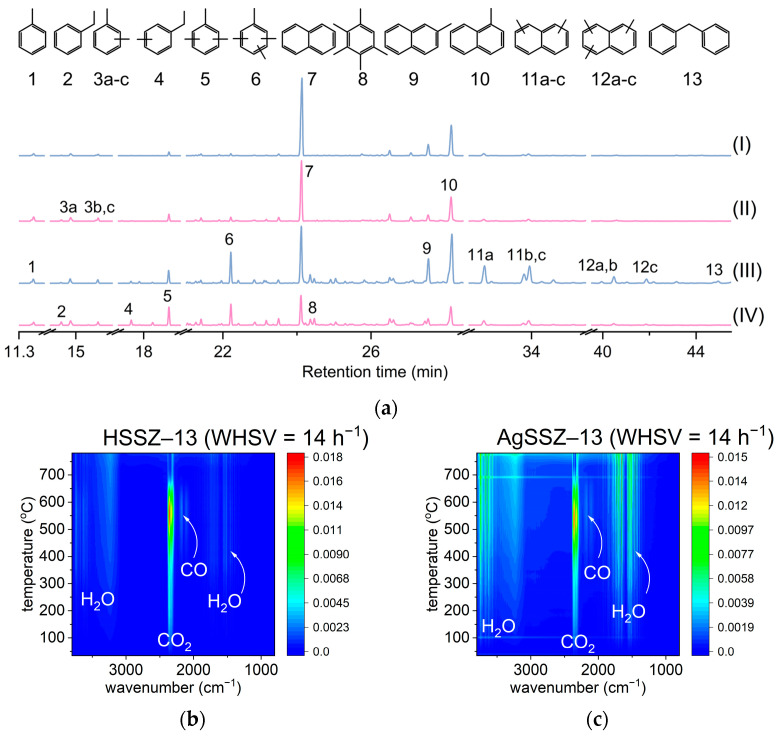
(**a**) GC-MS analysis of coke extracted from spent catalysts after *operando* FT-IR (I—HZSS-13, II—AgSSZ-13, WHSV = 7 h^−1^) and UV-vis (III—HSSZ-13, IV—AgSSZ-13, WHSV = 14 h^−1^) studies performed using different ethanol supplies; (**b**,**c**) FT-IR spectra of gas phase registered during the regeneration of spent catalyst inflow from synthetic air in the TG analysis.

A coke analysis was performed after both operando spectroscopic studies, applying different WHSV values; this allows the changes in the coke nature of different feed amounts to be followed. The coke analysis was performed after extraction from spent catalysts in the GC-MS analysis (Figure 4a). Further, the spent catalysts were analysed thermogravimetrically under synthetic air flow with a simultaneous record of the FT-IR spectra of effluent gases (Figure 4b,c).

The GC-MS results (Figure 4a) demonstrate that a low feed amount leads to the formation of more homogenous coke, with a dominant share of naphthalene and alkyl-substituted naphthalene. The coke from protonic and silver-loaded catalysts at lower feed amounts provides a similar picture; a slightly higher relative amount of substituted monoaromatics can be observed for silver-loaded catalysts. When the feed amount increases, resulting in a conversion drop, the coke species extracted from the spent catalysts are more heterogeneous in nature. First, a higher relative share of alkyl-substituted naphthalene is found in both catalysts. Similarly, tri- and tetraalkyl-substituted benzenes increase in relative content. Importantly, silver-loaded zeolite, presenting higher stability and selectivity to ethylene and propylene, has a much lower relative share of mono-, di-, and trialkyl-substituted naphthalene. The coke formed over the silver-loaded zeolite is composed of substituted benzenes and naphthalene with some share of monosubstituted naphthalene. For protonic catalysts among coke species, diphenylmethane was also identified.

The thermogravimetric analysis (Table 2), coupled with FT-IR spectroscopy of effluent gases, delivered further information on coke content and, tentatively, on coke nature and location; the coke content is distinctly higher for HSSZ-13, which is consistent with gas chromatography analysis and the results of *operando* FT-IR and UV-vis spectroscopic studies. The protonic zeolite exhibits lower stability during ethanol conversion, and the recorded spectra confirm the formation of heavy hydrocarbons as highly alkylated benzene and naphthalene. The AgSSZ-13 zeolite provides higher stability during the ethanol conversion, which is related to the accumulation of coke in lower amounts. The FT-IR spectra of the gas phase products of coke oxidation formed during the thermogravimetric analysis are presented in Figure 4b,c. For the HSZZ-13 sample, the coke oxidation products are composed mostly of CO_2_, and the highest CO_2_ elution is at 580 °C. The coke deposited over AgSSZ-13 zeolite is oxidised into CO_2_, CO, and H_2_O. The latter two gases are observable in the IR spectra. Thus, coke formed over AgSSZ-13 possesses a lower C/H ratio than coke from HSSZ-13 zeolite, and its oxidation proceeds at a lower temperature with the highest CO_2_ elution at 550 °C.

## 3. Materials and Methods

Commercially available SSZ-13 zeolite (Type A) with CHA topology (Si/Al = 8, H-form) was purchased from ACS Material LLC (Pasadena, CA, USA). A Ag-exchanged sample (AgSSZ-13) was obtained with an ion exchange procedure using an aqueous solution of AgNO_3_ (ACS reagent 99.9%) in the appropriate concentration to obtain 1.5 wt% using a solid/liquid ratio of 1/100 at 25 °C for 96 h under mechanical stirring and in darkness to avoid the reduction of Ag^+^ into Ag^0^. Then, the solids were filtered in darkness, washed with distilled water to remove excess silver, dried at 100 °C for 24 h, and stored in darkness. Both protonic and silver-exchanged zeolites were calcined at 550 °C before further examination.

The chemical composition of the catalysts was determined via inductively coupled plasma optical emission spectrometry (ICP-OES, Optima 2100DV, PerkinElmer, Waltham, MA, USA).

Powder X-ray diffractions were recorded on a Rigaku Multiflex diffractometer using Cu Kα radiation (40 kV, 40 mA).

The porosity was measured with nitrogen adsorption/desorption experiments using a Quantachrome Autosorb 1-MP gas sorption analyser. All samples were degassed at 10^−5^ mbar at 350 °C for 24 h before measurement. The specific surface area, S_BET_, was calculated using the Brunauer–Emmett–Teller method. The micropore volume (V_MICRO_) was calculated using the t-plot method. The differential micropore volume plot was calculated based on cylinder pore geometry (Saito–Foley).

Before the operando spectroscopic experiments, a catalyst self-supported disc (~25 mg) was placed in a 2 cm^3^ volume quartz IR cell (MeasLine, www.measline.com, accessed on 25 January 2024. patent PL232633, Cracow, Poland), where it was pre-treated under continuous helium flow for 60 min at 550 °C. The setup was then cooled to a reaction temperature of 325 °C. Then, helium as a carrier gas was passed over the liquid ethanol (analytical grade, 99% purity) containing the vessel, which was placed in a heating circulation bath (Huber, Germany) set at either 14 °C (WHSV = 7 h^−1^ for FT-IR) or 25.7 °C (WHSV = 14 h^−1^ for UV-vis). The total flow rate of the feed stream was kept at 40 cm^3^ min^−1^. The reaction run was investigated with an *operando* system connected to a flow setup. IR spectra were collected with a Vertex 70 Bruker spectrometer equipped with an MCT detector. The spectral resolution was of 2 cm^−1^. Analogously, for UV–vis (Shimadzu UV-2600, Shimadzu Corporation, Kyoto, Japan) experiments, the catalysts were prepared in a self-supported pellet form (~25 mg) and placed in a Praying Mantis^®^ cell. They were then pre-treated at 500 °C for 60 min. UV-vis spectra were recorded in operando mode at a reaction temperature of 325 °C. The products were simultaneously analysed via mass spectrometry (MeasLine, www.measline.com, accessed on 25 January 2024, RGA200, Cracow, Poland) and gas chromatography (Agilent Technologies 7890B, Agilent Technologies, Santa Clara, CA, USA).

Spent catalysts were grounded in an agate mortar and subjected to coke analysis. The total amount of coke was evaluated using TG-IR analysis with synthetic air flow. For this purpose, a portion of 5 mg of the catalyst in powder form was loaded into a 70 µL α-Al_2_O_3_ crucible and weighted with a NETZSCH STA 449 Fa5 Jupiter thermobalance (Netzsch-Gerätebau GmbH, Selb, Germany) before the analysis. The coke content of the spent catalysts was measured in TG experiments where the temperature was raised to 800 °C with a rate of 10 °C/min, and synthetic air flow was set at 80 mL/min. The outlet gas composition was examined via FT-IR spectroscopy, observing changes within H_2_O-, CO-, and CO_2_-corresponding bands.

To analyse species adsorbed on the catalyst surface, about 16 mg of used zeolite powder was placed in a Teflon vessel; then, 1 mL of 40% hydrofluoric acid, HF (Honeywell Fluka), was added, and the mixture was left for 1 h. Next, 6 mL of 20% boric acid, H_3_BO_3,_ was added, and after another hour, coke extraction was conducted using 1 g of dichloromethane (CH_2_Cl_2_). Thus, the extracted species were analysed with the aid of GC-MS measurements (Agilent Technologies 5977C, Agilent Technologies, Santa Clara, CA, USA).

## 4. Conclusions

The *operando* FT-IR and UV-vis spectroscopies are powerful tools for studying the process that occurs during ethanol conversion over HSSZ-13 and Ag-SSZ-13 zeolites. The coke analysis further supported the spectroscopic results, allowing for the correlation of the observed spectroscopic signatures with the species detected within the coke species. The formation of alkyl-substituted cyclopentenyl cations and alkyl-substituted benzenium ions was found based on UV-vis spectroscopic studies, and their mutual dependence was assumed to deactivate the catalysts. The FT-IR spectroscopic confirmed the formation of alkyl-substituted cyclopentenyl cations at earlier reaction stages. The protonic and silver-modified zeolites displayed distinctly different spectroscopic and catalytic characteristics, proving that the silver species modifies the activity and selectivity of chabazite in ethanol conversion.

## Figures and Tables

**Table 1:** The chemical composition (ICP), relative crystallinity, and textural parameters of the studied zeolite samples.

Sample	Si/Al	Ag wt%	Rel. Cryst. % ^1^	S_BET_ (m^2^·g^−1^)	V_MICRO_^2^ (cm^3^·g^−1^)
HSSZ-13	8.0	-	100	748	0.28
AgSSZ-13	8.3	1.6	90	755	0.28

^1^ Relative crystallinity (in %) was calculated as the ratio of the integral intensity of the most intense reflections within the 2Θ angle range from 22° to 27° to the sum of integral intensity for the protonic zeolite. ^2^ Micropore volume (*t*-plot method).

**Table 2:** The coke content (%) derived from TG experiments.

Sample	WSHV 7 h^−1^	WSHV 14 h^−1^
HSSZ-13	17.6	18.9
AgSSZ-13	9.2	12.4

## Data Availability

The raw data supporting the conclusions of this article will be made available by the authors upon justified and reasonable request.
